# Acute leukemias in pregnant women: Results of a retrospective study at a local tertiary‐care hospital in Japan

**DOI:** 10.1002/jha2.682

**Published:** 2023-04-05

**Authors:** Shuhei Kobayashi, Kyoko Biyajima, Shuji Matsuzawa, Kaoko Sakai, Fumihiro Kawakami, Toru Kawakami, Sayaka Nishina, Hitoshi Sakai, Chiho Fuseya, Hideyuki Nakazawa

**Affiliations:** ^1^ Department of Hematology and Medical Oncology Shinshu University School of Medicine Matsumoto Japan; ^2^ Department of Obstetrics and Gynecology Shinshu University School of Medicine Matsumoto Japan

**Keywords:** acute leukemia, multidisciplinary care, perinatal dilemma, pregnancy

## Abstract

Leukemia may rarely develop in a woman during pregnancy, posing clinical challenges to the patient, fetus, family, and medical staff managing malignancy and pregnancy. We retrospectively analyzed cases of pregnancy‐associated leukemia consecutively diagnosed and treated at a local tertiary‐care hospital in Nagano, Japan, over the past 20 years. Five cases were identified among 377,000 pregnancies in the area (one in every 75,000 pregnancies), all involving acute leukemia (three acute myelogenous leukemia [AML] and two acute lymphoblastic leukemia [ALL]). The cases were diagnosed in the first trimester (*n* = 1), second trimester (*n* = 3), or third trimester (*n* = 1). There were no apparent pregnancy‐associated delays in diagnosing and treating the cases. Three patients underwent induction chemotherapy during pregnancy, two of whom eventually delivered healthy babies. One of the five patients chose abortion before chemotherapy initiation. Two cases showing high‐risk features at the diagnosis (AML with an FLT3‐ITD mutation [*n* = 1] and relapsed ALL [*n* = 1]) eventually died despite consolidative allogeneic hematopoietic stem cell transplantation. Our results suggested that patients with pregnancy‐associated acute leukemia can be treated similarly to nonpregnant patients, although pregnancy imposes particular clinical challenges that should be resolved with multidisciplinary care.

## 1 INTRODUCTION

Acute leukemia is infrequently diagnosed during pregnancy, although the true incidence rate is unknown [1, 2, 3]. Based on previous reports published overseas in the 1980s, the incidence of such leukemia, both acute and chronic, was estimated to range from one in 75,000 to 100,000 pregnancies, [2] while a more recent report described the incidences as high as one in 10,000 pregnancies [4]. The majority of the cases of pregnancy‐associated leukemia are the acute type (acute myelogenous leukemia [AML] for two‐thirds and acute lymphoblastic leukemia [ALL] for one‐third of the cases), While up to 10% of cases are chronic myelogenous leukemia (CML) [1, 3]. Recently, the issue of fertility and pregnancy among CML patients of child‐bearing age has been receiving increasing attention, especially for patients treated with tyrosine kinase inhibitors [5, 6, 7, 8, 9].

Regardless of an acute or chronic nature, pregnancy‐associated leukemia frequently imposes particular clinical challenges on the patient, fetus, family, and multidisciplinary medical team helping manage the pregnancy, neonatal care, and malignancy [1, 10–13]. Furthermore, such clinical dilemmas are exacerbated when a patient presents with acute rather than chronic leukemia, as it will likely result in both maternal and fetal mortality in a relatively short period of time if left untreated [4, 13, 14]. In addition, while delaying the induction chemotherapy may negatively impact the likelihood of remission, patients and their families still need ample time and sufficient support to make their treatment decisions [4, 11].

Despite the above‐mentioned issues, a standard approach to addressing this clinical dilemma has not yet been established, partly due to the wide range of personal beliefs among patients [15]. Furthermore, the rarity of such cases also precludes large‐scale prospective controlled trials, and many recommendations in clinical guidelines regarding the management of pregnancy‐associated leukemia are derived from expert opinions [11, 16]. Although it has been suggested that standard chemotherapeutic intervention may help achieve a similar maternal survival rate to that in nonpregnant leukemic patients, a tailor‐made approach is mandatory to resolve potential and actual problems a pregnant patient may have to face, such as adverse effects on the pregnancy outcome, long‐term effects on the fetus, and impairment of the patient's future fertility [4].

We herein report the fetal and maternal outcomes as well as the dilemmas and their resolution in consecutive cases of pregnancy‐associated leukemias diagnosed and treated at a local tertiary‐care hospital the past 20 years. Our descriptions depict our perspective concerning rare but very challenging situations hemato‐oncologists may have to confront.

## 2 METHODS

### 2.1 Patients

Patients with pregnancy‐associated leukemia who were referred to and treated at the Department of Hematology and/or Department of Gynecology and Obstetrics of Shinshu University Hospital in Nagano, Japan, between 2001 and 2022 were enrolled. There were 44 medical offices and hospitals offering obstetrics services in Nagano Prefecture as of 2014 [17]. When a pregnant woman presents with a severe hematological abnormality, such as leukemia, in Nagano Prefecture, the patient is generally referred to Shinshu University Hospital, the only tertiary‐care hospital in the prefecture offering both obstetric service for high‐risk pregnancy, neonatology services, and adult hematology‐oncology services. The cohort of patients in the present study may therefore closely reflect the total incidence of pregnancy‐associated leukemia in this prefecture. Relevant clinical data on leukemia as well as the pregnancy and fetus were retrieved from patients’ medical records.

### 2.2 The diagnosis and treatment

The indication and procedures for bone marrow biopsies were the same in pregnant women as in patients without pregnancy when a leukemia was suspected. The diagnosis and classification of leukemia were confirmed in accordance with the WHO Classification Tumours of Haematopoietic and Lymphoid Tissues, 4th edition 2017 [18]. Radiographic evaluations, such as X‐ray and computed tomography (CT), were performed if necessary for differential diagnoses and evaluating complications, but all efforts were made to minimize potential radiologic exposure to the fetus. Cumulative anthracycline exposure dose was calculated by adding the doxorubicin‐equivalent dose of each anthracycline received. The conversion factors used to calculate doxorubicin equivalent were 1.0 for daunorubicin and 4.0 for mitoxantrone [19].

All patients were treated at the Department of Hematology, and their pregnancies were managed at the Department of Gynecology and Obstetrics of Shinshu University Hospital.

### 2.3 Statistical analyses

Vital statistics were obtained from a governmental database freely available online (e‐Stat), a portal site of official statistics of Japan managed by the Statistics Bureau [20]. The data included the annual local population as well as the total number of pregnant women in Nagano Prefecture. The incidence of leukemia was referenced from the Cancer Statistics issued by the National Cancer Center, Japan, a registry dataset also freely available online [21].

### 2.4 Ethical considerations

Leukemia patients who did not agree to be included in the present study were excluded from the cohort using an opt‐out method. This study was approved by the institutional review board of Shinshu University School of Medicine (approval number: 5756, February 14, 2023) and conducted in accordance with the Declaration of Helsinki.

## 3 RESULTS

### 3.1 Patients

We identified and enrolled five patients with pregnancy‐associated acute leukemia during the study period: three AMLs and two ALLs (Table 1). There were no cases with chronic leukemias nor acute promyelocytic leukemia. The median age (range) was 33 (19–40) years old at the diagnosis of leukemia. The pregnancies were gravida 1 and para 0 in all the five cases. The medical histories were insignificant in four cases, but one patient had been in a state of complete remission for 3 years after chemotherapy for ALL. The symptoms at presentation were variable, including skin and mucosal bleeding in two cases, a low‐grade fever in one case, and a reactivation of varicella zoster infection in one case. The remaining patient was asymptomatic, but a hematological abnormality was identified incidentally during a regular health check for pregnant women. A bone marrow examination was performed in all five cases as safely as in leukemia patients without pregnancy.

**TABLE 1:** Five cases of pregnancy‐associated acute leukemia.

				At presentation						
UPN	Age (yeas old)	Pregnancy	Medical history	Symptom	WBC (/µL)	Blast (%)	Hb (g/dL)	plt (x10^4^/µL)	Diagnosis of leukemia	Gestational week at diagnosis of leukemia
#1	40	G1P0	NA	Varicella Zoster	18,850	47	12.7	7.9	AML	26 weeks 4 days
#2	19	G1P0	NA	Low grade fever	7870	53	6.1	7.0	AML with RUNX1	16 weeks 5 days
#3	33	G1P0	Urethrolithiasis	Gingival bleeding lower back pain	16,800	95	10.4	1.1	Ph positive ALL	33 weeks 6 days
#4	33	G1P0	NA	Pethachea	13,930	83	11.5	2.4	AML with FLT3‐ITD	11 weeks 6 days
#5	36	G1P0	Ph negative ALL	Asymptomatic	17,450	86	8.9	2.1	Ph negative ALL	15 weeks 5 days

Abbreviation: AML, acute myelogenous leukemia; UPN, Unique Patient Number; WBC, white blood cell; .

According to the e‐Stat, there were 377,000 pregnancies in Nagano Prefecture, Japan, between 2001 and 2022, so we estimated the incidence of pregnancy‐associated acute leukemia to be 1.33/100,000 pregnancies, or 1 in 75,000 pregnancies in Nagano (Supplemental Table). Nagano Prefecture had a population of two million, including 244,000 women between 20 and 44 years old as of 2019 [20]. Registry data retrieved online showed that the incidence of leukemia, including chronic and acute cases, among the general female population between 20 and 44 years old varied with age between 2.5/100,000 and 4.5/100,000 in 2019 [21].

### 3.2 Management of pregnancies and neonatological care

**FIGURE 1:**
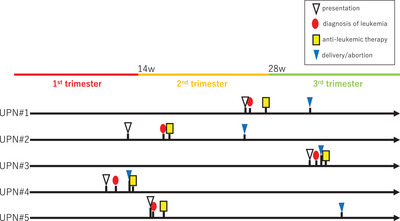
Clinical courses of pregnancy in patients with pregnancy‐associated leukemia are depicted. Each horizontal black bar represents a patient, with four time‐points, each indicating the day of diagnosis of acute leukemia (red circle), the day of initial presentation (white triangle), the day of initiation of induction chemotherapy (yellow rectangular), and the day of delivery or abortion (blue triangle). The colored bar at the top represents three trimesters of pregnancy.

Leukemia was diagnosed in the first trimester in one patient, second trimester in three patients, and third trimester in one patient (Figure 1). The patient whose gestation was in the first trimester (UPN#4) selected abortion before the initiation of anti‐leukemic treatment as opposed to continuing the pregnancy throughout the treatment. The three patients in the second trimester proceeded with the induction chemotherapies with the intention to continue their pregnancy until the gestational week of 32 or longer, in order to avoid high neonatal morbidity and mortality associated with respiratory immaturity, which would be expected in cases of very preterm birth earlier than 32 gestational weeks [22]. Intrauterine chemotherapeutic exposure of the fetus was eventually observed in 3 patients (Table 2). One of the patients (UPN#1) received a diagnosis of acute leukemia at gestational week 26 and started chemotherapy at gestational week 27. She eventually underwent Caesarean section at gestational week 32 and delivered a healthy baby. Another patient (UPN#5) was found to have a relapsed leukemia at gestational week 15 and began reinduction chemotherapy at week 16 of gestation. She experienced a preterm premature rupture of membranes at gestational week 34 during a chemotherapy session, and her delivery of a healthy baby was uneventful. The other patient (UPN#2) had her leukemia diagnosed at week 16 of gestation and started induction chemotherapy in week 17 of gestation. However, in gestational week 25, during one of the consolidation chemotherapies with high‐dose cytarabine, oligohydramnios was diagnosed, necessitating emergent Caesarean section because of fetal distress. A still birth was diagnosed, and the hemoglobin level of the umbilical cord was 2.9 g/dL. The patient who received her leukemia diagnosis in her third trimester underwent emergent Caesarean section at gestational week 33 because of fetal distress. She eventually delivered a healthy baby and proceeded to undergo induction chemotherapy.

**TABLE 2:** Outcomes of pregnancy‐associated leukemia and the fetus.

	Maternal treatment and outcome of leukemia							fetus		
						Intrauterine exposure to cytotoxic agents				
UPN	Induction chemotherapy	Response to induction	Postinduction therapy	Cumulative anthracycline dose (mg/m^2^)*	Survival Outcome**		Anthracycline dose (mg/m^2^)***	Cytarabine dose (g/m^2^)***	Delivery or abortion	Survival at one month
#1	7+3 induction	CR	consolidation with anthracyclin and cytarabine	484	Alive 2 months	YES	250	0.7	32w+5 Caesarean section live birth	Alive
#2	7+3 induction	CR	consolidation with high‐dose cytarabine	250	Alive 8 months	YES	250	20.7	26w+0 Caesarean section stillbirth	NA
#3	Dasatinib + steroid	CR	allo‐HSCT	180	Alive 24 moths	NO	0	0	34w+1 Caesarean section live birth	Alive
#4	7+3 induction	CR	allo‐HSCT	234	Dead 21 months	NO	0	0	13w+0 abortion	NA
#5	Hyper CVAD	CR	consolidation with CHOP followed by allo‐HSCT	418	Dead 52 months	YES	250	0	34w+6 vaginal delivery live birth	Alive

Abbreviations: CR, complete remission; HSCT, hematopoietic stem cell transplantation; Hyper CVAD, hyperfractionated cyclophosphamide, vincristine, doxorubicin, and dexamathasone; UPN, Unique Patient Number; WBC, white blood cell.

*Cumulative anthracycline exposure dose was calculated by adding the doxorubicin‐equivalent dose of each anthracyline received. The conversion factors used to calculate doxorubicin equivalent were 1.0 for daunorubicin and 4.0 for mitoxantrone. **Survival outcome represents a time period from the diagnosis of pregnancy‐associated leukemia to the time on the last confirmed day. ***Intrauterine exposure dose of cytotoxic agents was displayed per maternal body surface area.

### 3.3 Management of leukemias

The median (range) duration from the initial presentation to the diagnosis of leukemia was 7 (0–28) days, and the median (range) duration from the diagnosis to the start of cytotoxic chemotherapy was 7 (6‐12) days. Standard induction chemotherapy was eventually administered in all five patients; cytotoxic chemotherapy preceded delivery in three patients, while anti‐leukemic treatments followed an abortion in one patient and emergent Caesarean section in another (Figure 1, Table 2). All patients achieved complete remission (CR) after induction chemotherapies. The median (range) dose of cumulative anthracycline was 250 (180–484) mg/m^2^. The two ALL patients eventually proceeded to allogeneic hematopoietic stem cell transplantation (allo‐HSCT); the patient who underwent transplantation during the first CR, remained alive at 24 months from the diagnosis of leukemia (UPN#3), while the other who received transplantation at the second CR, experienced a second ALL relapse 6 months after the transplant (UPN#5). One of the AML patients harboring an FLT‐ITD mutation received allo‐HSCT as postinduction therapy, but the disease relapsed 6 months after the transplant. Another patient with a RUNX1‐RUNX1T1 has been in molecular remission for 3 months since the completion of consolidation chemotherapy (UPN#2). Consolidative therapies are ongoing in the other patient with AML with an intermediate risk (UPN#1). With a median (range) time of observation of 21 (2–52) months, three are alive with CR, and two were dead from complications after second or third allo‐HSCT.

The indication of red blood cell transfusions for patients with pregnancy‐associated leukemia was the same for leukemia patients without pregnancy; a hemoglobin level of 7 g/dL was considered a trigger of transfusion as described in a guideline issued by the Ministry of Health, Labour and Welfare in Japan [23]. The trigger for platelet transfusions during pregnancy was decided after careful discussion between obstetricians and hematologists: 20 to 30 × 10^3^/µL during pregnancy, 50 × 10^3^/µL for vaginal delivery, and 80 × 10^3^/µL for Caesarean section. Additional transfusions were also performed based on obstetric indications. There were no severe hemorrhagic complications in any patients.

### 3.4 Management of perinatal dilemmas

Multidisciplinary discussions and consultations were frequently held as needed for each case to identify and resolve medical problems as well as ethical dilemmas, such as conflicts of interest between the mother and fetus [24]. Supportive talk sessions with the patients and their families were repeated as requested, even after critical informed consent was obtained. Any disagreement on treatment plans among physicians and other medical staff were resolved through discussions. Detailed descriptions of representative perinatal dilemmas identified in our cohort of pregnancy‐associated leukemia and a result of multidisciplinary discussions to resolve the dilemmas are summarized in the Appendix.

## 4 DISCUSSION

We identified a total of five cases of pregnancy‐associated acute leukemia occurring in Nagano Prefecture in the past 20 years, suggesting an estimated incidence of 1.33/100,000 pregnancies or 1 in 75,000 pregnancies in this region. We did not find any cases of adult T‐cell leukemia (ATLL) with pregnancy in our cohort, largely because the prefecture is located in less‐endemic area for HTLV‐1 [25, 26]. It is generally accepted that, unlike ATLL, geographical distribution of other acute leukemias is not significantly different among prefectures in Japan, so our results may accurately reflect the incidence of pregnancy‐associated acute leukemia in Japan as a whole. This frequency, as well as the ratio of myeloid and lymphoid leukemia, was comparable to the findings in previous studies conducted overseas [2]. Our results also suggest that pregnancy‐associated acute leukemia is rare in Japan and that pregnancy was not associated with an increased frequency of leukemia.

The small size and short observation period of our study hamper the drawing of any hard conclusions concerning the prognostic impact of pregnancy on the clinical course of leukemia. However, it may be worth mentioning that we did not note any significant delays in the diagnosis or initiation of treatment of leukemia due to pregnancy itself. In a previous study of malignant lymphoma during pregnancy, the start of chemotherapy was deferred in approximately 40% of cases [27]. Our findings may reflect the fact that any delay in the start of induction therapy for acute leukemia may negatively impact on both the patient and their fetus [4, 14] and may also reflect the urgent nature of this clinical situation. Our results may also confirm that a standard anti‐leukemia treatment strategy may be carefully sought even during pregnancy [4, 11, 13, 15].

Short‐term adverse effects were not observed in either of the two live‐born children who experienced intrauterine exposure to cytotoxic chemotherapies. Our results may confirm previous observations that standard treatment options should be offered to patients with pregnancy‐associated leukemia, as they would be in patients without pregnancy [28]. However, the AML patient with high‐dose cytarabine consolidation chemotherapy who suffered a still birth intensified discussion of the clinical dilemma among treating hematologists about whether or not it was ethically appropriate to offer high‐dose therapy to the patient, which might have jeopardized the fetal survival. Although there was no clear association between the fetal distress and the high‐dose chemotherapy itself, the severe anemia of the fetus was attributed to myelosuppression due to intrauterine exposure to maternal chemotherapy, including high‐dose cytarabine [29]. Notably, the hematologists involved all agreed that this patient with AML would have a better overall survival rate with core binding factor with high‐dose therapy than with standard‐dose consolidation chemotherapy [30, 31]. However, although careful discussion was repeated, and informed consent was obtained from the patient, such misgivings lingered among the staff, which may represent a part of the challenge encountered in cases of pregnancy‐associated acute leukemia. The two patients with dismal outcomes had already had significant risk factors at the time of the diagnosis of pregnancy‐associated leukemia: an FLT3‐ITD mutation in the AML patient and relapsed Ph‐negative ALL, both of which are known to confer a poor prognosis among adult patients [32–37]. Their poor outcomes may therefore not have been associated with their pregnancy.

In summary, pregnancy‐associated acute leukemia was treated in the same manner as leukemia in patients without pregnancy. Multi‐disciplinary care is mandatory for patients in such challenging situations, but special clinical dilemmas should be resolved in sessions of supportive discussion with patients as well as among staff members throughout the treatment period.

## FUNDING INFORMATION

The authors received no specific funding for this work.

## CONFLICT OF INTEREST STATEMENT

The authors have no conflict of interest to be disclosed.

## ETHICS STATEMENT

Informed consent of participating patients was obtained using an opt‐out method. This study, including this opt‐out method, was approved by an institutional review board of Shinshu University School of Medicine, approval number 5756, approval date February 14th, 2023. And this study was conducted in accordance with the Declaration of Helsinki. The statement of IRB approval is described in the Method section.

## Supporting information

Supp Information

## Data Availability

The data that support the findings of this study are available from the corresponding author upon reasonable request.

## 1

A representative case of a challenging perinatal dilemma

A 19‐year‐old pregnant woman (UPN#2) presented to our hospital with a persistent low‐grade fever. Her pregnancy was 16 weeks and 5 days of gestation, and she was otherwise a healthy expectant mother. The laboratory data showed bicytopenia with 53% myeloid blasts in the bone marrow, so a diagnosis of acute myelogenous leukemia (AML) was made. Her cytogenetic analysis showed that her leukemia harbored the t(8;21)(q22;q22.1) and RUNX1‐RUNX1T1 mutations. Careful and supportive discussion between the patient, her family, and her treating hematologists and obstetricians were repeated, even after informed consent was obtained. The patient decided to receive standard induction chemotherapy during her pregnancy. She eventually achieved complete remission (CR) after her first round of induction chemotherapy.

Because of a feature of the core binding factor (CBF) leukemia, she was offered the option to receive three courses of consolidative chemotherapy with high‐dose cytarabine, which was expected to provide patients with CBF leukemia a significantly better disease‐free survival (DFS) rate (57%) than the conventional 4 courses of consolidation (39%) (*p* = 0.050) [31]. However, there was a different but compelling opinion among the treating physicians that such a benefit had not translated into a better overall survival (OS) in the relevant clinical trial (75% vs. 66%, *p* = 0.174) and that the intensive post‐induction chemotherapy might jeopardize the survival of the fetus more than less‐intensive consolidative therapy. The patient and her family eventually chose high dose‐cytarabine treatment. Her leukemia was in CR after completion of the first course of the intensive therapy, but she required emergent Caesarean section because of fetal distress. Unfortunately, she had a stillbirth, and the baby had a very low hemoglobin level (2.9 g/dL). Although there was no clear association between the fetal distress and the high‐dose chemotherapy itself, the severe anemia of the fetus was attributed to myelosuppression due to intrauterine exposure to maternal chemotherapy. The patient completed three courses of consolidation therapy and was discharged home with her disease in molecular remission.

***A result of multidisciplinary discussions on the representative case (UPN#2)***

A multidisciplinary conference was held for this case almost once a week at a hospital ward, involving treating physicians, residents, hematologists, nurses, nurse specialists, pharmacists, a nutritionist. Discussions were also frequently held with obstetricians in different occasions. Informal discussions among these staff, as a curbside‐consultation manner, were also held countlessly throughout the clinical course of the patient.

All team members were expected to share the pertinent clinical information of the patient, regarding the diagnosis, treatment options, expected results, and adverse effects in each option, expected prognosis in each option, patient's character and preferences, social background of the patient and available support from her family members. Social workers also input necessary information to the team members at their request. Any concerns or dilemmas were encouraged to be addressed by team members, either formally during a conference or informally during a curbside, and we tried to identify medical, ethical or emotional issues behind the concerns in straightforward terms.

Our multidisciplinary discussions concluded that one of the medical challenges as ethical dilemmas we encountered in this case was a conflict of interest between the expectant mother and the fetus [24]. It is generally accepted that suboptimal treatment of the mother's condition may harm the fetus as well as the mother. The treating physicians acknowledged the concerns about the risks the high‐dose consolidation therapy posed to the fetus but eventually concluded that such concern for the fetus should not lead to inappropriately withholding more‐effective treatment from the mother. Furthermore, the patient and her family understood and consented to the advice. There was, however, an equally compelling question from some staff members about whether or not the benefit of pursuing a better DFS rate of 57%, instead of 39%, without a significant OS benefit was worth risking the fetus, although we all agreed that the very‐high‐dose chemotherapy was not the sole cause of the fetal distress. All staff members also agreed that there would not be a single perfect answer to solve this clinical dilemma, and that repeated multidisciplinary discussions and shared‐decision‐making process with patient and family is crucial.

This case may be representative of the clinical challenges facing patients with pregnancy‐associated acute leukemia and the multidisciplinary teams treating such patients in similar situations. Our results suggested that repeated discussions among staff members as well as the patient can help resolve such lingering misgivings.
